# Food insecurity and its consequences in indigenous children and youth in Canada

**DOI:** 10.1371/journal.pgph.0002406

**Published:** 2023-09-27

**Authors:** Anna Banerji, Veronique Anne Pelletier, Rodney Haring, James Irvine, Andrew Bresnahan, Barry Lavallee

**Affiliations:** 1 Pediatrics, University of Toronto, Toronto, Ontario, Canada; 2 Toronto General Hospital, University of Toronto, Toronto, Ontario, Canada; 3 Dalla Lana School of Public Health, University of Toronto, Toronto, Ontario, Canada; 4 Consultant Pediatrician, General Pediatrics, CHU Ste-Justine, Montreal, QC, Canada; 5 Department of Pediatrics, University of Montreal, Montreal, QC, Canada; 6 Academic Family Medicine and Community Health and Epidemiology, University of Saskatchewan, Saskatoon, Saskatchewan, Canada; 7 Chair, Roswell Park Department of Indigenous Cancer Health, Roswell Park Comprehensive Cancer Center, Buffalo, New York, United States of America; 8 Adjunct Faculty, Frost Centre for Canadian Studies and Indigenous Studies, Trent University, Peterborough, Ontario, Canada and Iqaluit, Nunavut, Canada; 9 Keewatinohk Inniniw Minoayawin Inc., Winnipeg, Canada; Western University Schulich School of Medicine & Dentistry, CANADA

## Abstract

Food insecurity (FI) is at a crisis level in some Indigenous communities and impacts many of the half million First Nations Inuit and Métis (FNIM) children across Canada, particularly in isolated northern communities. This can lead to malnutrition and can have significant impacts on the physical, intellectual, emotional and social development of a child, often with lasting effects across the life course. This is a narrative review article with extensive search of the medical literature with input from the FNIM National organizations. The primary cause of FI is an imbalance between the high price of food relative to household income, where poverty is a driving factor. The cost and lack of availability to healthy foods has resulted in a transition to unhealthy market foods. Food security programs need to be prioritized, multi-faceted and multi-tiered within a framework of food sovereignty. Translational science, research, to practice is also important. The use of successful Indigenous based models of FI, towards food sovereignty using self-determination, Indigenous Knowledge, strength-based models, and ancestral sustainability are critical. Continued community-based evaluation of FI towards sustainable healthy food programs are important for communities to initiate track, evaluate, and grow robust community-based programs to counter-balance FI. Continued scientific research in the fields of FI, food sovereignty, and their relationship to co-occurring conditions related to healthy eating and beverage consumption are vastly important to the health of Indigenous Peoples. These are all part of many Indigenous connection to the earth, through food source, the maintenance of health through ancestral ways of living, set in the premise of looking forward multiple generations towards the continued resiliency through food, diet, relationship, and sovereignty. Food Security is a human right and needs to be urgently addressed for Indigenous children in Canada.

## Introduction

The World Food Summit in 1996 declared that food security occurs, “when all people, at all times, have physical, social and economic access to sufficient, safe and nutritious food that meets their dietary needs and food preferences for an active and healthy life” [1]. Food security is critical for the physical, intellectual and social development of a child [2] and is related to a child’s ability to recover from illness, linked to school performance and important for the prevention of chronic disease [3, 4]. Unfortunately, there is severe food insecurity (FI) among many First Nations (FN), Inuit and Métis (FNIM) families and communities across Canada. According to the United Nations Declaration of Human Rights [5], food security is a human right. However, for many of the nearly 500,000 Indigenous children in Canada (approximately 65% FN, 30% Métis and 5% Inuit) [6] adequate access to safe and nutritious food is not being met. The extent and severity of FI in these communities makes it an urgent public health issue [7–11].

## Methods

The goal of this narrative review is to answer the question *“What is the extent of food insecurity in Indigenous Children in Canada*. *The sub-questions are “What are the contributing factors and what are possible solutions for food insecurity in Indigenous communities in Canada*?*”* This review was compiled through the following approach: with review of peer-review literature, review of reports from First Nations, Inuit, and Métis (FNIM) National organizations, [Assembly of First Nations (AFN), Inuit Tapiriit Kanatami (ITK), and the Métis National Council (MNC)], as well as advice for recommendations to address FI from the National FNIM organizations during stakeholder meetings.

This paper is a narrative review of publications searched in the following data base: Medline, Pubmed, Google Scholar; reports published by the three national Indigenous Organisations grey literature and reports from FNIM organisations [12–15]. The publications selected for this review had to include the following key words: food insecurity or food security, Indigenous or Inuit or Métis or First Nations, children or youth and Canada. The analysis and extraction of the data for the main question were completed separately for Inuit, First Nations and Métis. Examples of solutions used for food security in Indigenous Communities and their children are presented however these are not analysed. The goal of this review is to inform policy at government levels, community and individual levels, to help ameliorate this complex problem of food insecurity in Indigenous children in Canada.

For the purpose of this article, Indigenous refers to First Nations, Inuit, and Métis populations in Canada, where the term Aboriginal is only used if it is part of a title. Abbreviations and terminology are recorded in S1 Text.

### Scope of the problem

According to Statistics Canada, moderate FI is defined as inadequate quality and/or quantity of food consumed, and severe FI encompasses problems of missed meals, reduced food intake, and possibly whole days without food [11, 16, 17]. FI is a Canada wide-problem. However, it is at crisis levels for many Indigenous populations, specifically in areas of Canada’s North with high proportions of Indigenous Peoples [11, 16–19]. A direct comparison of the prevalence of FI within and among Indigenous groups, and with the overall Canadian population is challenging as the surveys vary in the study population (northern, urban, remote, on and off-reserve), age groups, criteria for FI, and methodology. The data show a disproportionately higher rate of moderate to severe FI in all Indigenous groups. Rural and remote Indigenous communities in the North are at a particularly high risk [20], although FI impacts Indigenous Peoples living on and off reserve including urban regions [14, 21–24]. Specific (FNIM) regions further illuminate the scope of FI among Indigenous children in Canada.

### Inuit children

The Nunavut Inuit Child Health Survey in 2007–2008 assessed for FI preschool Inuit children aged 3–5 in 16 Arctic communities. Alarmingly 69.6% of households had FI (34.4% severe and 35.3% moderate) [7]. Of the severely food insecure homes, children skipped meals (75.8%) went hungry (90.4%) or did not eat for a full day (64.3%) in the past year due to insufficient money for food [10]. The 2012 Aboriginal Peoples’ Survey found that 53% of Inuit over the age of 15 in Inuit Nunangat (Inuit homelands) lived in households which experienced FI [25] and documented that 52% of Inuit older than 25 years of age living in the Arctic lived in households which experienced FI: Nunavut 55%, Nunavik 55%, Nunatsiavut 42% and Inuvialuit Settlement Region 33% [26]. In Iqaluit, the capital of Nunavut, FI was identified in 32.9% of households with children compared to 23.2% of households without [27]. The odds of FI among adolescents were higher in Nunavut (aOR 6.77), Northwest Territories (aOR 2.11) compared to Ontario [28]. Food security in Inuit Nunangat is a public action priority for ITK, Inuit land claims organizations, and governments, as indicated in the Inuit Nunangat Food Security Strategy [29].

### First nations children

The National FN Regional Health Survey documented that over half of FN adults were moderately or severely FI. For those households with children, 43% were FI [30]. The First Nations Food, Nutrition and Environment Study (FNFNES) across-Canada participatory study of 92 on-reserve First Nations (south of 60° from 2008 to 2018) in partnership with the AFN, documented that 54% of households with children experienced FI vs 36% without children [23] and made recommendations to address FI [13]. In a FN Territory in Northern Ontario the prevalence of FI was 76% in households with children [20].

### Métis children

There is a deficiency of data regarding FI for Métis children and more research is required. Métis children present a high prevalence of risk factors for food insecurity [31, 32]. Métis children are more likely to live in larger households, are twice as likely to live in a single parent household, and twice as likely to live in poverty compared to non-Aboriginal children, all risks factors for FI [14]. According to the Métis National Council (MNC), the lack of access to land (through historical dispossession) for harvesting traditional foods and agriculture, has been a long-standing issue for FI. The MNC’s Virtual Dialogue on Food Security and the Métis Nation in 2021 provided a summary of Métis FI issues and strategies [15].

While Indigenous populations in remote areas have a high prevalence of FI, it is an issue for urban Indigenous children as well. Métis and off-reserve FN youth in Saskatchewan and Ontario struggled to have healthy foods due to the unaffordability, availability, and lack of proximity without transportation [22, 33]. They resorted to energy dense market food (MF), as it was cheaper and lasted longer. The authors concluded that food insecurity manifested itself in different ways and suggest that obesity prevention strategies should take a family-targeted approach that consider the unique barriers facing urban Indigenous Peoples.

### Determinants of FI

The contributing factors for FI in Indigenous populations are complex and include individual and community factors, historical, social and political factors. Batal described a paternalistic system that systematically provides inferior care, with a higher prevalence of malnutrition and obesity [34]. The Truth and Reconciliation Commission’s 2015 report [35] documented that trauma from residential schools and other discriminatory policies that have led to inter-generational trauma, addictions, underemployment and poverty for some individuals and communities, all of which contribute to FI [36]. Adults who attended or those with a parent or a grandparent who attended a residential school had a higher proportions of severe FI than those who did not (16.4%, 16.2% respectively verses 6.9%) [21]. The legacies of colonization, including many treaties, the Indian Act, and residential schools were strongly tied to policies focused on the assimilation and control of Indigenous People [37, 38]. Some of these policies impacted the availability and accessibility of food such as buffalo, and restricted hunting rights with confinements to reserve areas. Some cultural sharing practices involving feasts and ceremonies were outlawed, resulting in loss of inter-generational knowledge of traditional food procurement and preparation [38].

The pervasive driver for FI is poverty [11]: the high cost of market food (MF) which is commercially produced foods and is transported in, compared to the household income. Families more vulnerable to FI include those: with children, of larger size, single parent households headed by women, with lower education attainment, on income assistance or with limited income, limited knowledge of healthy MF, living in overcrowded conditions, and living in remote communities [8, 11, 24, 39]

### Traditional foods and market foods

Over thousands of years, Indigenous populations have adapted to a diet suitable to their environment. Traditional (country) foods (TF) included any animals and plants harvested from the local environment [39]. The type of TF consumed reflected the local environment. For example, for coastal communities, fish and marine animals were a predominant part of the diet, berries and land animals in the interior. In Arctic regions above the tree line with a very short growing season, meats, fish, and marine animals have long been important TF [39].

TF were healthy and had all the nutrients required for a healthy active life, while food shortages occurred at times. TF tend to have higher amounts of protein, healthy fats, vitamins and minerals as well as lower amounts of saturated fat, carbohydrates, sugars and sodium [15] than most available MF. Along with the nutritional benefits of TF, hunting and gathering of TF is commonly a community activity that required physical activity and is deeply rooted in cultural traditions and identity, including networking and sharing [39].

Indigenous Peoples in Canada have gone through rapid change from traditional land-based food production to acculturation and participation in the wage economy where MF have become a greater part of the diet for many Indigenous communities [40]. The high cost of nutritious MF, especially in remote areas in comparison to household income, is a major contributor of FI. The 2017 Nunavut Food Price Survey compared the cost of staple food items demonstrated that the cost of food in Nunavut was at least double that of the rest of Canada [41]. Surveys through FNFNES also demonstrated FI across Canada where remoteness was correlated with food costs. In the most northern FN communities of Saskatchewan, a FNFNES survey revealed food costs almost triple those in the southern urban center [42].

With the social demographic changes in lifestyle, MF which are commercially produced foods have been transported in and are now a major portion of the diet [40]. The high cost of hunting including the cost of transportation, gasoline, ammunition, and lack of hunting equipment are major contributors to the demographic change [39, 43, 44]. Historically TF were shared with the extended households and the community, especially to those families who did not have a hunter, as having an active hunter in the family is associated with reduced FI [40, 45]. The sale of TF is an increasing trend in many areas. While potentially increasing access to TF for some, it risks limiting the exchange to those who can afford it, reproducing income-mediated patterns of access to healthy foods [39].

Environmental regulations and jurisdictional issues can also be barriers to the community’s ability to hunt on their traditional territories. Climate change, changing the migratory routes of animals, and declines in some animal populations such as caribou, walrus, changes the accessibility, availability and quality of TF and has significant dietary implications [40, 46]. Food safety concerns from naturally occurring or man-made contaminants have the potential of decreasing use of TF, and increasing FI, [47–49]. Persistent organic pollutants (POP) such as organochlorines, polychlorinated biphenyl (PCB)s and heavy metals, such as mercury and lead tend to accumulate in Arctic and sub-Arctic regions [40, 47, 48] and bioaccumulate in the food chain, especially in larger animals such as toothed whales, seal and walrus. Despite this, TF continues to be recognized by elders as healthier than MF, and important in the prevention of illness [39, 48, 49]. Traditional food consumption was associated with adequate nutrient intakes in Nunavut Inuit children aged 3–5 years [50]. Ideally a balance of TF and/or healthy MF needs to be optimized and combined to prevent FI, especially when TF is less available.

Displacing TF with high energy and nutrient poor MF, such as sweetened drinks or chips, tends to increase excess calories, especially from fat and carbohydrates, contributing fewer vitamins and minerals [51]; however, MF is more affordable. Long transportation distances and delays often result in perishables being of poor quality, expired, expensive or unavailable [43]. In Inuit communities in Nunavut, non-nutrient-dense foods were acquired three times more frequently than more nutrient-dense foods [52]. Studies in Indigenous communities including Inuit, Dene and Métis communities found that the younger generation preferred MF over TF with diets that had higher carbohydrate intake mostly in the form of sucrose and a trend towards greater saturated fats [53] but were inadequate in calcium, Vitamin A, folic acid and fiber.

### Consequences of FI

FI in a child can have numerous adverse short-term and potential life-long consequences on their health, education and future employment. FI emerges as a predictor of multiple forms of malnutrition such as undernutrition, being overweight and obesity [54]. Malnourished children are more likely to subsequently develop acute and chronic illnesses including cardiac disease, diabetes and cancer [11, 55, 56]. FI in young children is associated with lower cognitive development outcomes and behavioral problems [2, 3]. Hunger also impedes learning as children have difficulty trying to learn while hungry and can experience reduced academic achievements [2]. FI in childhood has also been associated with future unemployment, and can perpetuate the vicious cycle of poverty [2, 3]. It can create stress in the family environment and contributes to anxiety and depression [3, 57]. FI has been associated with poor general and mental health, high stress levels, and life dissatisfaction [58] and an increased risk of dysthymia and thoughts of suicide in adolescents [59]. Severe household FI in Nunavik in childhood and adolescence was associated with persistent psychological distress in adolescence [57]. For youth living in Inuit Nunangat from 1994 to 2008, the mortality rate in Inuit children was 5 times, and suicide rate 30 times higher than the national Canadian average respectively [60].

Micronutrient deficiencies in Indigenous children, related to FI have been associated with numerous adverse health outcomes. FI in Nunavik was associated with nutritional deficiencies especially iron deficiency anemia (IDA), and FI children were an average of 2 cm shorter than food secure children [61]. IDA was identified in 36% of children in a surveillance of Indigenous children in the James Bay area [62]. Vitamin D-deficient rickets which is associated with FI (55) disproportionately impacts Indigenous and northern children in Canada with an incidence of rickets in 1 and 2 year old children in the three Canadian territories equal to 6–12 times higher than the rest of Canada [63, 64].

Dental caries surveys in Inuit and FN, reported very high rates in children: from 69 to 90%, with an average of 6–8 teeth with dental decay by 12–19 years [65]. The risk was associated with increased consumption of soda pop (sweetened beverages), and the lack nutritional supplements containing Vitamin D, calcium or fluoride, and not drinking milk [51]. This was all compounded by poor access to oral healthcare [65, 66]. Poor diet prior to conception can have lasting effects on the infant’s health. In eastern James Bay, Northern Quebec, in an area with low levels of maternal dietary folate, there is a high prevalence of spinal bifida [67].

Obesity can be a significant consequence of FI along with its related health issues [68]. FI was associated with an elevated BMI in Inuit households in the Arctic [11]. Indigenous children are twice as likely to be classified as obese [69]. From the Aboriginal People’s Survey assessing youth ages 6 to 17, 22% of Métis and FN were overweight and 15% obese [69]. Households with very high FI were at higher risk of being overweight or obese. In 18 Canadian Inuit communities, there was a significant decrease in TF and increase in MF, with an increase in BMI over a decade from 1998 [70]. In Cree children grades 4–6 in northern Quebec, 29.9% were overweight and 34.4% were obese and Cree children aged 9–18, 67.6% of were overweight where the majority of the diet consisted of high energy dense MF such as sweetened beverages and snack foods with intakes of zinc, calcium and vitamin D below the recommended intake [71]. The lack of clean water in many Indigenous communities has resulted in the substitution of water with sugary drinks (soda, juice) contributing to the obesity issue [72]. One of the most serious consequences of obesity is the epidemic of type 2 diabetes in many FN communities [73, 74] as well as cardiac disease.

## Discussion

As presented in this narrative review, FI for Indigenous Peoples is the result of a complex interplay of social, economic, political, historical, geographical factors. Mitigating FI will take a concerted holistic effort with partners at all levels of government and continuous engagement of communities. While strategies to increase access to healthy foods is important to alleviate FI among Indigenous communities, poverty is a predominant factor for FI and there is an urgent need for poverty reduction strategies [11]. All FI strategies need to be Indigenous-led, and embedded within a framework of *Food Sovereignty*, which is the right of nations and people to control their own food systems, markets, production, food culture and environments [19, 40].

The Nutrition North Canada (NNC) strategy was initiated in 2011 by Indigenous and Northern Affairs Canada, to make nutritious and perishable food more accessible and affordable to Canadians living in isolated northern communities. The subsidy is provided directly to northern retailers, food suppliers and distributors. Concerns have been raised about the subsidy not being transferred to the consumer, the lack of accountability by retailers to the federal government and the criteria for community selection [75–77].

Several examples of food security strategies have been implemented in different contexts (Table 1) with the objective of having access to an adequate supply of safe, culturally preferable, affordable, nutritious TF and MF which promotes environmental sustainability, from community led breakfast programs with TF and healthy MF, to community kitchens, gardens and greenhouses as well as community freezers, subsidizing ammunition as well as poverty reduction strategies (Table 1). The FN community of Kahnawà:ke demonstrated a process of reclaiming food security through sustainable self-determination, traditional knowledge, and Indigenous principles [74, 78]. In 2021, the three Indigenous organizations ITK, AFN and MNC made recommendations for food security [13, 15, 29], related to their context. Indigenous Peoples have always maintained a connection to TF. Although the connection, relationship, and use may have changed due to the changing landscape following foreign government-to-Indigenous government relationships, land movement, and foreign cultural dietary influences. Indigenous Knowledge of food systems is still strong among many Indigenous communities [12, 78]. As such, the re-connection to ancestral foods, healthy sustenance farming, and traditional foraging, are important strengths of Indigenous People, including children and youth [12, 78].

**Table 1:** Examples of indigenous food security strategies in Canada.

Program	Brief description
Nutrition North Canada (NNC)	Initiated in 2011 by Indigenous and Northern Affairs Canada, the purpose is to make nutritious and perishable food more accessible and affordable to Canadians living in isolated northern communities. A subsidy is provided directly to northern retailers, food suppliers and distributors. However, concerns have been raised about the subsidy not being transferred to the consumer, the community selection criteria and the lack of accountability https://www.nutritionnorthcanada.gc.ca/eng/1415385762263/1415385790537
Canada Prenatal Nutrition Program (CPNP)	Funds community groups to develop or enhance programs for vulnerable pregnant women to, improve the health of both infant and mother and encourage breastfeeding. https://cpnp-pcnp.phac-aspc.gc.ca/en
School breakfast programs ONEXONE, Breakfast Clubs Canada and Feed the Children.	Partnered with Indigenous communities across Canada to include: Community led breakfast programs with community leadership and participation with TF and healthy MF, as determined by the community, (Gillies C, Blanchet R, Gokiert R, Farmer A, Thorlakson J, Hamonic L, et al. School-based nutrition interventions for Indigenous children in Canada: a scoping review. BMC Public Health. 2020 2020/01/06;20(1):11) [81]
Aboriginal Food Security in Northern Canada: An Assessment of the State of Knowledge	Comprehensive report on the contributors of FI and strategies for improving access for healthy foods for Indigenous populations in Canada with a list of Canadian resources. https://cca-reports.ca/wp-content/uploads/2018/10/foodsecurity_fullreporten.pdf
The Northern Healthy Foods Initiative (NHFI)	Supports a variety of northern food security and food sovereignty initiatives in 70 rural areas in Manitoba. https://home.cc.umanitoba.ca/~thompso4/NHFIfinal2010.pdf
First Nations Food, Nutrition and Environment Study (FNFNES)	Collected data from 2008 to 2018 across many FN communities in Canada south of the 60^th^ parallel providing information on FI and influences of FI, nutritional quality of the diet, use of TF, and environmental contamination of TF. https://www.fnfnes.ca/
Nunavut Food Security Coalition	Collaborative action plan that lists strategies for country food, store-bought food, local food, life skills, program and community initiatives, and policy and legislation with the objective of having access to an adequate supply of safe, culturally preferable, affordable, nutritious food through a food system that promotes Inuit Societal Values, self-reliance, and environmental sustainability.” https://www.nunavutfoodsecurity.ca/node/933
Inuit Tapiriit Kanatami. An Inuit-Specific Approach for the Canadian Food Policy.	An Inuit-specific approach for food policy with diverse stakeholders to develop strategies to improve food systems in the Canadian Arctic,
Inuit Tapiriit Kanatami, *Inuit Nunangat Food Security Strategy*.	Focuses on availability, accessibility, quality and lists strategies for country food, store-bought food, local food, life skills, program and community initiative, policy and legislation
The Northern Food Network (NFN)	Co-hosted by the Arctic Institute of Community-Based Research (AICBR) and Food Secure Canada (FSC) as a space for people working in and interested in northern food security to share, learn about best practices across the North and advance collective action on food security.
Community Greenhouses such as Inuvik, Gjoa Haven, Île-à-la-Crosse,	Inuvik has had a successful year-round community garden/greenhouse for over 20 years. This model could be expanded to all northern communities so they can grow fresh produce and provide employment, (https://www.inuvikgreenhouse.com/). Other examples include Gjoa Haven, and Île-à-la-Crosse (https://www.ctvnews.ca/canada/anything-is-possible-nunavut-greenhouses-bring-food-jobs-to-tundra-1.4787362, https://www.cbc.ca/news/canada/saskatchewan/students-solar-power-greenhouse-1.6127063)
Food Security & the Métis Nation: Métis National Council	Métis National Council had a virtual dialogue with Métis Leaders to discuss strategies for improved food security and food sovereignty June 15 2021

### Recommendations

Food security is a Human Right [1]. In view of the many specific risk factors and the highest prevalence in Canada, FI must be addressed as an urgent public health crisis for Indigenous Peoples in Canada. In addition, we must respect and incorporate the United Nations Declaration on the Rights of Indigenous Peoples (UNDRIP) which acknowledges the rights of Indigenous Peoples “to engage freely in all their traditional and other economic activities”, including traditional hunting and harvesting [79].

### Strategies and policies

Taking into considerations that Indigenous Peoples and their children in Canada are overrepresented amongst those affected by FI, the specificities relating to the consequences of the colonialism, a National Indigenous Food Strategy Program within a food sovereignty framework are required. Key components include involvement of community leadership, recognizing community and individual food preference, and increasing the availability and quality of TF and healthy MF. It must be resourced to ameliorate, monitor, and evaluate FI strategies [19].

### a) Addressing poverty

As the primary driver for FI is poverty, especially in relation to the high cost of healthy MF and the ability to obtain TF, poverty reduction programs are critical to reducing FI in Indigenous populations in Canada [11]. Coordinated poverty reduction strategies involving Indigenous organizations, territorial, provincial and federal governments [11] need to be developed. This includes income support to reflect the real cost of living and increasing funding for programs supporting FNIM employment. Prioritizing a reform of the Nutrition North Program into an evidence-based food security program, increasing community eligibility, subsidizing healthy foods, hunting and harvesting, with adequate oversight and accountability will help to ensure subsidies result in better access and quality of MF and TF.

### b) Translation into practice: FNIM-led food strategies

Translational science research to practice is very important. It is imperative to support and implement the recommendations of FNIM-led food security strategies, including the FNFNES (2021), MNC’s report on Food Security & the Métis Nation (2021), and ITK’s Inuit Nunangat Food Security Strategy (2021) [12–14] which outline clear objectives and actions for improving food security and poverty reduction strategies. Other measures include:

- Enabling the acquisition of healthy TF by subsidizing ammunition, and gas for hunters, and support community harvesting and storage community freezers, school and traditional harvesting programs [12, 13, 19].
- Maintaining and improving harvester support programs support community wellness and intergenerational knowledge sharing and include land-based education and healing as well as sustainable wildlife management [13, 14, 19].
- Ensuring universal child access to healthy foods through federal funding for established school-based and early childhood nutrition and education programs serving Indigenous communities need to be continued and expanded to communities where they do not yet exist [80].
- Promoting food production enterprise opportunities within Indigenous communities, increase self-sufficiency and increase employment opportunities, and innovations such as greenhouses which grow fresh produce (Table 1).

### c) Surveillance, monitoring and research

There is an urgent need to address the many gaps in knowledge with high quality research especially where there is a paucity of data, in areas such as urban Indigenous and Métis children, food safety and contamination and and others identified by the Indigenous organizations. Continued research in the fields of FI, food sovereignty, and their relationship to co-occurring conditions related to healthy eating and beverage consumption, and monitoring food safety with respect to environmental toxins in the food chain, are vastly important to the health of Indigenous Peoples. Continued evaluation of FI towards sustainable healthy food programs are important for communities to initiate track, evaluate, and grow robust community-based programs to counter-balance FI. There must be adequate funding for the high-quality, multi-year surveillance data for FI and monitoring FI reduction programs, especially in vulnerable communities in Canada and in particlar for training of Indigenous scholars. Quality Improvement interventions with community leadership, and partership with qualified research teams, are desirable, and could led to more rapid changes and improvements [81].

### d) For the clinicians

Clinicians should consider early screening for food insecurity in communities or families at risk. Validated evaluation tools for food insecurity in children are available to the clinicians and require basic clinical communication skills [68, 82]. However, these would need to be validated culturally with the different Nations. The clinician should also be aware of resources available to access quality food in sufficient quantity (such as community organizations, food banks and others). Collaboration with a dietician and/or a traditional knowledge keeper are also excellent resources to have at the clinic, community health center, or hospital. Vitamins and micronutrients supplementation should be provided as needed, when documented or at risk of deficiency in pregnant mother and/or in children. Beyond the immediate health needs, clinicians are well advised to collaborate upstream with the community leaders and public health.

### e) Honoring indigenous knowledge

Lastly and most important, is the transition away from deficit-based models such as “Food Insecurity “towards strength-based models, such as Indigenous Knowledge. By doing so, it embraces culturally focused strength-based food sovereignty programs which incorporate and honor ancestral ways of life, language, governance, and traditions. These are all part of many Indigenous connection to the earth, through food source, the maintenance of health through ancestral ways of living, set in the premise of looking forward multiple generations towards the continued resiliency through food, diet, relationship, and sovereignty.

## Conclusion

There is a FI crisis in many Indigenous communities in Canada. This can lead to malnutrition and can have significant impacts on the physical, intellectual, emotional, and social development of a child, often with lasting effects across the life course. The use of successful Indigenous based models of FI, towards food sovereignty using self-determination, are integral. Food security programs need to be prioritized, multi-faceted and multi-tiered starting with the premise that food security is a human right.

## Supporting information

S1 Text Terminology and abbreviations. (DOCX)
