# Correction: Food insecurity and its consequences in indigenous children and youth in Canada

**DOI:** 10.1371/journal.pgph.0003552

**Published:** 2024-07-22

**Authors:** The PLOS GLOBAL PUBLIC HEALTH Staff

The affiliation for the third and fourth author is incorrect. Rodney Haring is not affiliated with #6 but with #7: Chair, Roswell Park Department of Indigenous Cancer Health, Roswell Park Comprehensive Cancer Center, Buffalo, New York, United States of America. James Irvine is not affiliated with #7 but with #6: Academic Family Medicine and Community Health and Epidemiology, University of Saskatchewan, Saskatoon, Saskatchewan, Canada.

The publisher apologizes for the error.

## References

[1] Banerji A, Pelletier VA, Haring R, Irvine J, Bresnahan A, Lavallee B (2023) Food insecurity and its consequences in indigenous children and youth in Canada. PLOS Glob Public Health 3(9): e0002406. 10.1371/journal.pgph.0002406 37756390 PMC10530329

