# Supplementary material for: Food insecurity and its consequences in indigenous children and youth in Canada
Source: PLOS Glob Public Health. 2023 Sep 27;3(9):e0002406. doi: 10.1371/journal.pgph.0002406 (PMC10530329; doi:10.1371/journal.pgph.0002406)
Supplement: S1 Text — (DOCX) [file pgph.0002406.s001.docx]

S1 Text: Terminology and Abbreviations

AFN: Assembly of First Nations

BMI: Body Mass Index

FI: Food insecurity

FN: First Nations

FNIM: First Nations, Inuit and Métis

FNFNES: First Nations Food, Nutrition and Environment Study

IDA: iron deficiency anemia

ITK: Inuit Tapiriit Kanatami

MF: Market Food

MNC: Métis National Council

Nunangat: Inuit homelands in Canada

TF: Traditional Food

UNDRIP: United Nations Declaration on the Rights of Indigenous Peoples
